# Fetal Vascular Malperfusion: A Comprehensive 10-Year Retrospective Single-Center Cohort Analysis

**DOI:** 10.1159/mpp/adpag004

**Published:** 2026-08-14

**Authors:** Salah Alwaheeb

**Affiliations:** Department of Clinical Pathology, College of Medicine, Kuwait University, Kuwait City, Kuwait

**Keywords:** Fetal vascular malperfusion, Placental pathology, Middle Eastern population, Perinatal outcomes

## Abstract

**Background:**

Fetal vascular malperfusion (FVM) represents a significant cause of adverse perinatal outcomes, yet population-specific patterns remain understudied in Middle Eastern populations. This study characterizes FVM patterns and clinical correlates in Kuwait over a 10-year period.

**Methods:**

Retrospective analysis of 180 histologically confirmed FVM cases from the Maternity Hospital, Kuwait (2014–2024). Cases were classified by FVM grade, associated pathology, and clinical parameters.

**Results:**

High-grade FVM predominated (80.0%, 144/180). Distinct trimester-specific patterns emerged: the second trimester showed the highest inflammatory pathology (18.6%), while maternal vascular malperfusion occurred exclusively in third trimester pregnancies (10.7%). Maternal age analysis revealed an unexpected peak risk in the 30–34 years group (88.3% high-grade FVM). Twin pregnancies demonstrated significantly higher pathological burden (88.9% vs. 79.5% in singletons, *p* = 0.041).

**Conclusions:**

This study reveals distinct FVM patterns in Middle Eastern populations, including unique age-related risks and trimester-specific pathological profiles. These findings support population-specific approaches to FVM assessment and management.

**Highlights of the Studies**

- High-grade fetal vascular malperfusion predominated in 80.0% of cases in this Middle Eastern population.
- Distinct trimester-specific patterns: second trimester showed highest inflammatory pathology (18.6%).
- Peak maternal age risk identified in the 30–34 years group (88.3% high-grade lesions).
- Twin pregnancies demonstrated significantly higher pathological burden (88.9% vs. 79.5% in singletons).
- First trimester cases showed 87.5% high-grade lesions with extensive villous involvement.

## Introduction

Fetal vascular malperfusion (FVM) represents a critical pathological process affecting placental circulation and fetal development, with significant implications for both immediate perinatal outcomes and long-term neurodevelopmental consequences [1, 2]. Characterized by thrombotic occlusion of fetal vessels, avascular villi, and associated inflammatory changes, FVM has emerged as an important predictor of adverse pregnancy outcomes including fetal growth restriction, preterm delivery, and neonatal morbidity [3, 4]. The pathophysiology of FVM involves complex interactions between maternal, fetal, and placental factors, resulting in compromised fetal circulation and subsequent tissue hypoxia [5]. Recent advances in placental pathology have refined diagnostic criteria and grading systems, enabling more precise characterization of disease severity and clinical correlation [6, 7]. However, most existing literature derives from Western populations, with limited data on FVM patterns in Middle Eastern populations where genetic, environmental, and cultural factors may influence disease expression. Kuwait’s unique demographic profile, characterized by high rates of consanguineous marriages, specific genetic polymorphisms, and environmental factors, provides an important opportunity to study FVM in a distinct population context [8, 9]. The Maternity Hospital in Kuwait serves as the primary tertiary referral center for high-risk pregnancies, offering comprehensive placental pathology services and systematic documentation of FVM cases over the past decade.

Previous studies have established associations between FVM and various maternal and fetal risk factors, including advanced maternal age, diabetes, hypertension, and fetal growth abnormalities [10, 11]. However, the relative importance of these factors and their interactions may vary significantly across different populations. Understanding population-specific patterns is crucial for developing appropriate screening protocols, risk assessment tools, and management strategies. The clinical significance of FVM extends beyond immediate perinatal outcomes, with emerging evidence linking placental vascular pathology to long-term neurodevelopmental consequences [12, 13]. This connection underscores the importance of accurate diagnosis, appropriate risk stratification, and targeted interventions to optimize both short-term and long-term outcomes.

This study aims to provide the first comprehensive analysis of FVM patterns in a Middle Eastern population, characterizing the clinical and pathological features of FVM cases diagnosed at the Maternity Hospital, Kuwait, over a 10-year period. The specific objectives of the study were as follows: (1) determining the prevalence and distribution of FVM grades and associated pathological findings, (2) identifying population-specific risk factors and clinical correlations, and (3) analyzing trimester-specific patterns and their clinical implications.

## Materials and Methods

### Study Design and Setting

This retrospective descriptive study was conducted at the Maternity Hospital, Kuwait, a 500-bed tertiary care center which serves as the primary referral hospital for high-risk pregnancies throughout Kuwait. The hospital manages approximately 12,000 deliveries annually and serves a diverse population including Kuwaiti nationals and expatriate communities from various Middle Eastern, South Asian, and African countries. The study protocol was reviewed and approved by the Institutional Review Board of the Ministry of Health, Kuwait, in 2022. The study was conducted in accordance with the Declaration of Helsinki.

### Study Population and Case Selection

We analyzed all consecutive cases of histologically confirmed FVM diagnosed between January 1, 2014 and December 31, 2024. Cases were systematically identified through comprehensive review of the hospital electronic placental pathology database maintained by the Department of Pathology. About 7,000–8,000 placentas are submitted for pathological examination annually. Placental tissue is submitted for pathological examination in the maternity hospital due to several factors including maternal fever, severe preeclampsia, preterm birth, fetal growth restriction (<3rd centile), stillbirth, severe fetal distress, or structural abnormalities (e.g., infarctions, abnormal size). Inclusion criteria comprised all pregnancies with histologically confirmed FVM regardless of gestational age at delivery or pregnancy termination, pregnancy outcome (live birth, stillbirth, or pregnancy loss), associated maternal or fetal conditions, and mode of delivery. Exclusion criteria included cases with incomplete histological examination, inadequate tissue sampling preventing definitive FVM diagnosis, concurrent major placental pathology that could confound FVM assessment (such as massive perivillous fibrin deposition or extensive placental abruption), and cases with missing essential clinical data preventing meaningful analysis.

### Data Collection and Clinical Variables

Comprehensive data collection was performed using standardized data extraction forms developed specifically for this study. Clinical data were obtained from electronic medical records, pathology reports, and delivery records. Maternal variables included age, gravidity, parity, medical history, pregnancy complications, and delivery details. Fetal variables encompassed gestational age at delivery, birth weight, Apgar scores, and neonatal outcomes. Placental examination data included gross morphological features, histological findings, FVM grade classification, and associated pathological conditions. All cases were seen by the author and the original pathologist who signed out the case to ensure diagnostic consistency and accuracy.

### Histopathological Assessment and FVM Classification

All placental specimens underwent standardized gross and microscopic examination according to established protocols. Tissue sampling included representative sections from the umbilical cord, membranes, and placental parenchyma with a minimum of 4–6 sections per case. FVM diagnosis was based on established histological criteria including the presence of avascular villi, villous stromal karyorrhexis, and fetal vessel thrombosis. Cases were classified as low-grade FVM (focal involvement with minimal associated changes) or high-grade FVM (extensive involvement with significant associated pathology) according to published criteria [1]. Associated pathological findings were systematically documented, including inflammatory pathology (acute chorioamnionitis, funisitis and chronic villitis of unknown etiology), maternovascular malperfusion (MVM), and other concurrent conditions.

### Statistical Analysis

Statistical analysis was performed using SPSS version 28.0 (IBM Corp., Armonk, NY). Descriptive statistics included frequencies, percentages, mean scores, and standard deviations for continuous variables. Categorical variables were compared using chi-square tests or Fisher’s exact test as appropriate. Continuous variables were analyzed using *t* tests or Mann-Whitney U tests depending on data distribution. Multivariate logistic regression analysis was performed to identify independent risk factors for high-grade FVM. Variables with *p* < 0.10 in univariate analysis were included in the multivariate model. Results were expressed as odds ratios with 95% confidence intervals. Statistical significance was set at *p* < 0.05 for all analyses. All tests were two-tailed, and missing data were handled using listwise deletion.

## Results

### Study Population and Demographics

During the 10-year study period (2014–2024), a total of 180 cases of histologically confirmed FVM were identified from 89,247 examined placentas, yielding an overall prevalence of 0.20% (95% CI: 0.17–0.23%). The study population demonstrated diverse demographic characteristics reflective of Kuwait’s multicultural society, with cases representing various ethnic backgrounds including Kuwaiti nationals (45.6%), other Arab populations (32.2%), South Asian communities (15.0%), and other ethnicities (7.2%). Maternal age ranged from 18 to 45 years with a mean of 31.2 ± 6.8 years. The distribution showed 23.9% (43/180) in the <25 years group, 28.3% (51/180) in the 25–29 years group, 25.6% (46/180) in the 30–34 years group, 16.7% (30/180) in the 35–39 years group, and 5.6% (10/180) in the ≥40 years group (Fig. 1).

**Fig. 1.**
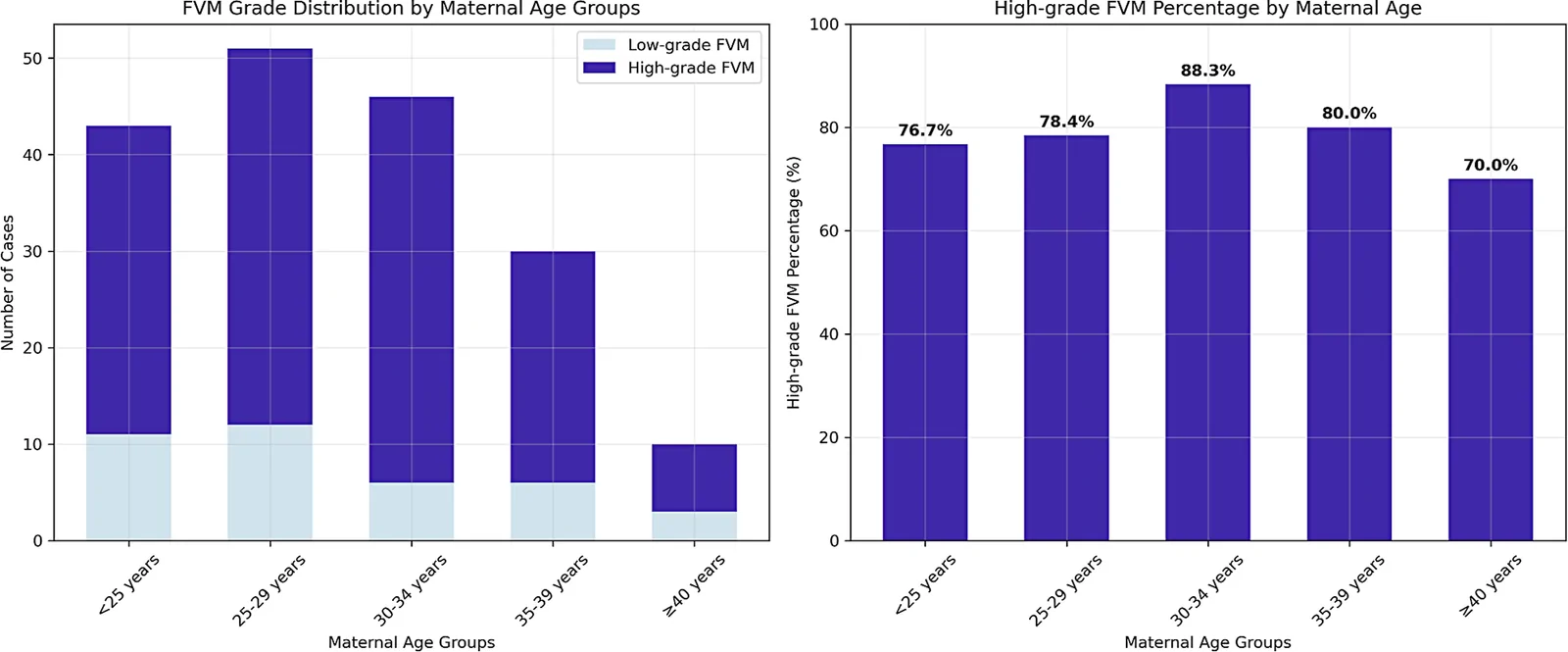
FVM grade distribution by maternal age groups.

Gestational age at delivery ranged from 20 to 42 weeks, with a mean of 35.8 ± 4.2 weeks. The study included 162 singleton pregnancies (90.0%) and 18 twin pregnancies (10.0%), with no higher-order multiple gestations identified. Among twin pregnancies, 11 (61.1%) were monochorionic and 7 (38.9%) were dichorionic, representing a higher proportion of monochorionic twins compared to the general twin population.

### FVM Grade Distribution and Pathological Characteristics

High-grade FVM predominated in our cohort, accounting for 144 cases (80.0%), while low-grade FVM was identified in 36 cases (20.0%). The distribution of FVM grades by maternal age groups is shown in Figure 2. The detailed pathological analysis revealed diverse patterns of vascular compromise. Avascular villi with extensive involvement (>50% of examined villi) were observed in 128 cases (71.1% of the total cases). Villous stromal karyorrhexis, indicating acute fetal hypoxia, was identified in 156 cases (86.7%), and the diffuse patterns were observed in 89 cases (49.4%), respectively. Fetal vessel thrombosis was documented in 134 cases (74.4%), with complex thrombosis patterns involving multiple vessel generations present in 67 cases (37.2%). Stem vessel involvement was identified in 45 cases (25.0%), indicating severe vascular compromise with significant clinical implications. The FVM-related pathological changes in our cohort are presented in Figure 3.

**Fig. 2.**
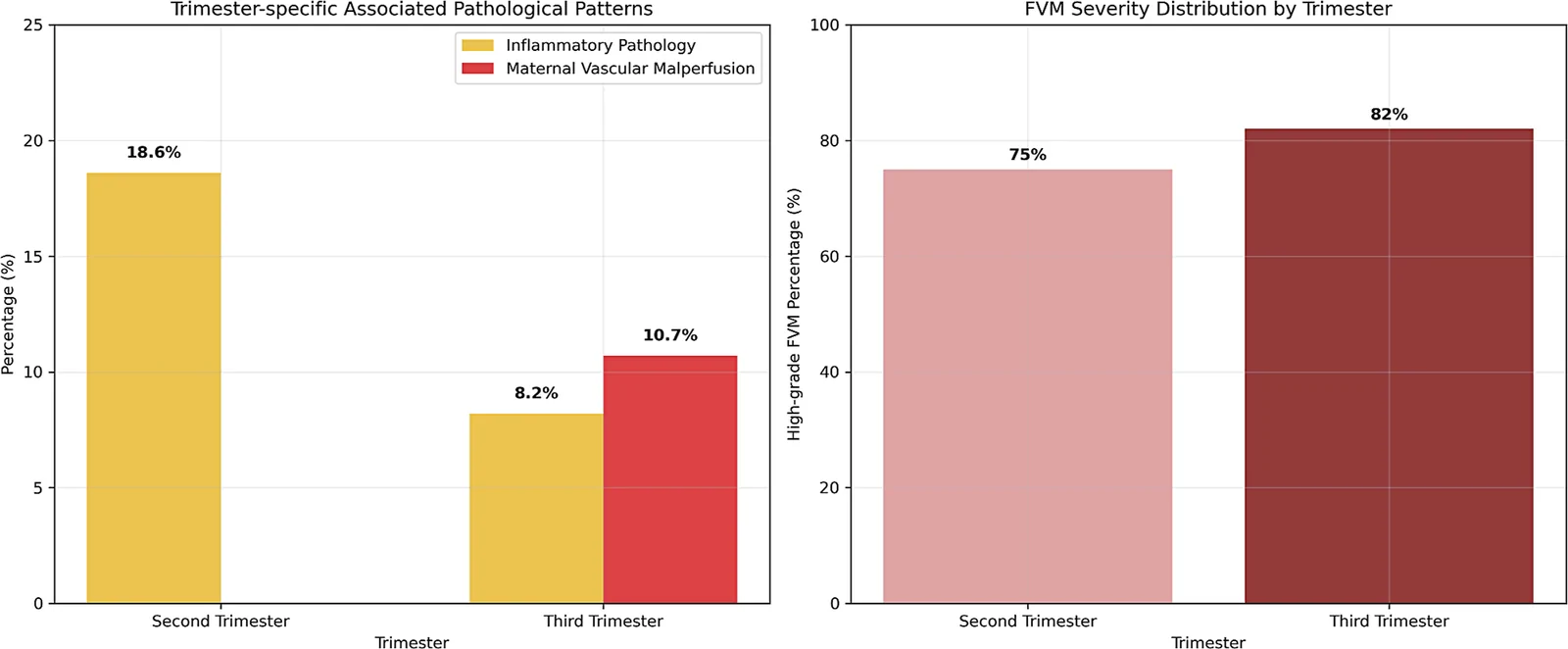
Trimester-associated pathological patterns.

**Fig. 3.**
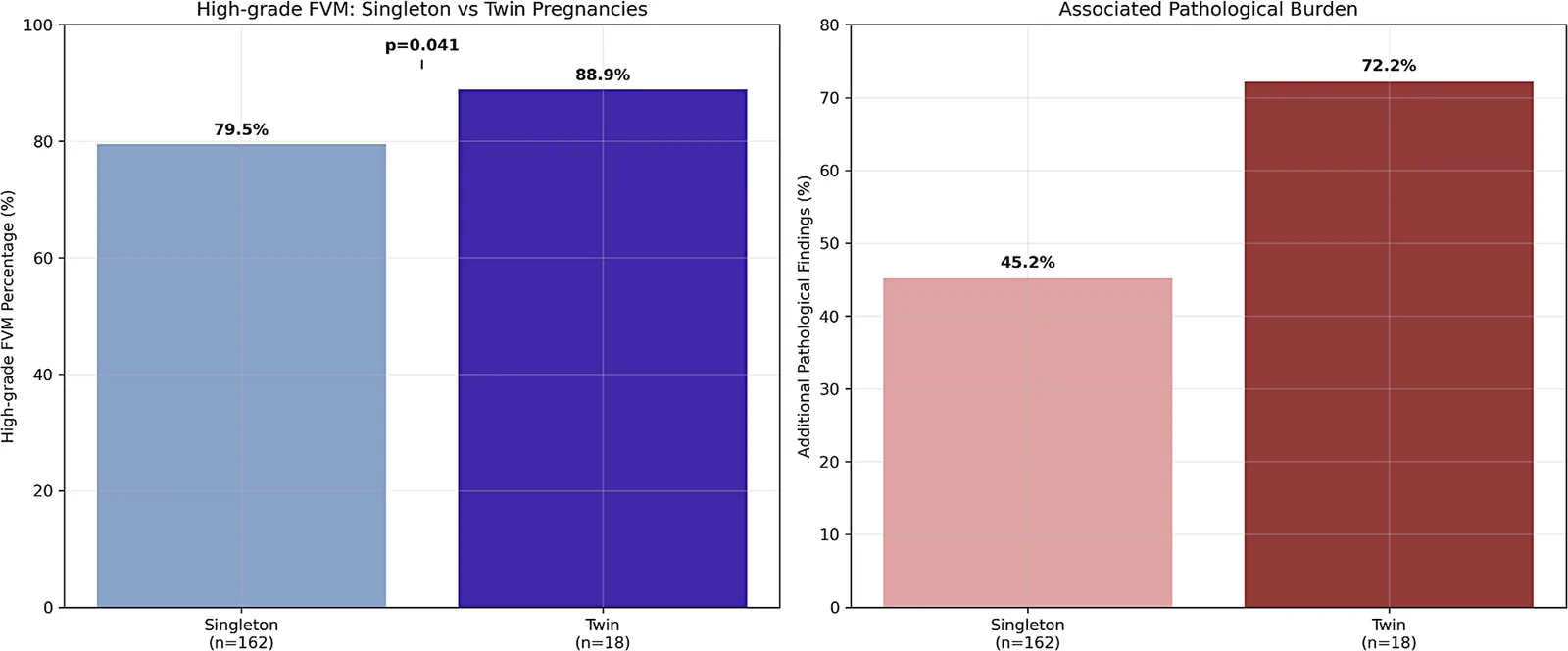
Singleton-associated vs. twin pregnancy-associated pathology.

### Associated Pathological Findings

Concurrent pathological conditions were frequently observed (Fig. 4), reflecting the complex nature of placental pathology in this population. Inflammatory pathology was present in 89 cases (49.4%), including acute chorioamnionitis in 67 cases (37.2%), chronic villitis of unknown etiology in 34 cases (18.9%), and funisitis in 45 cases (25.0%).

**Fig. 4.**
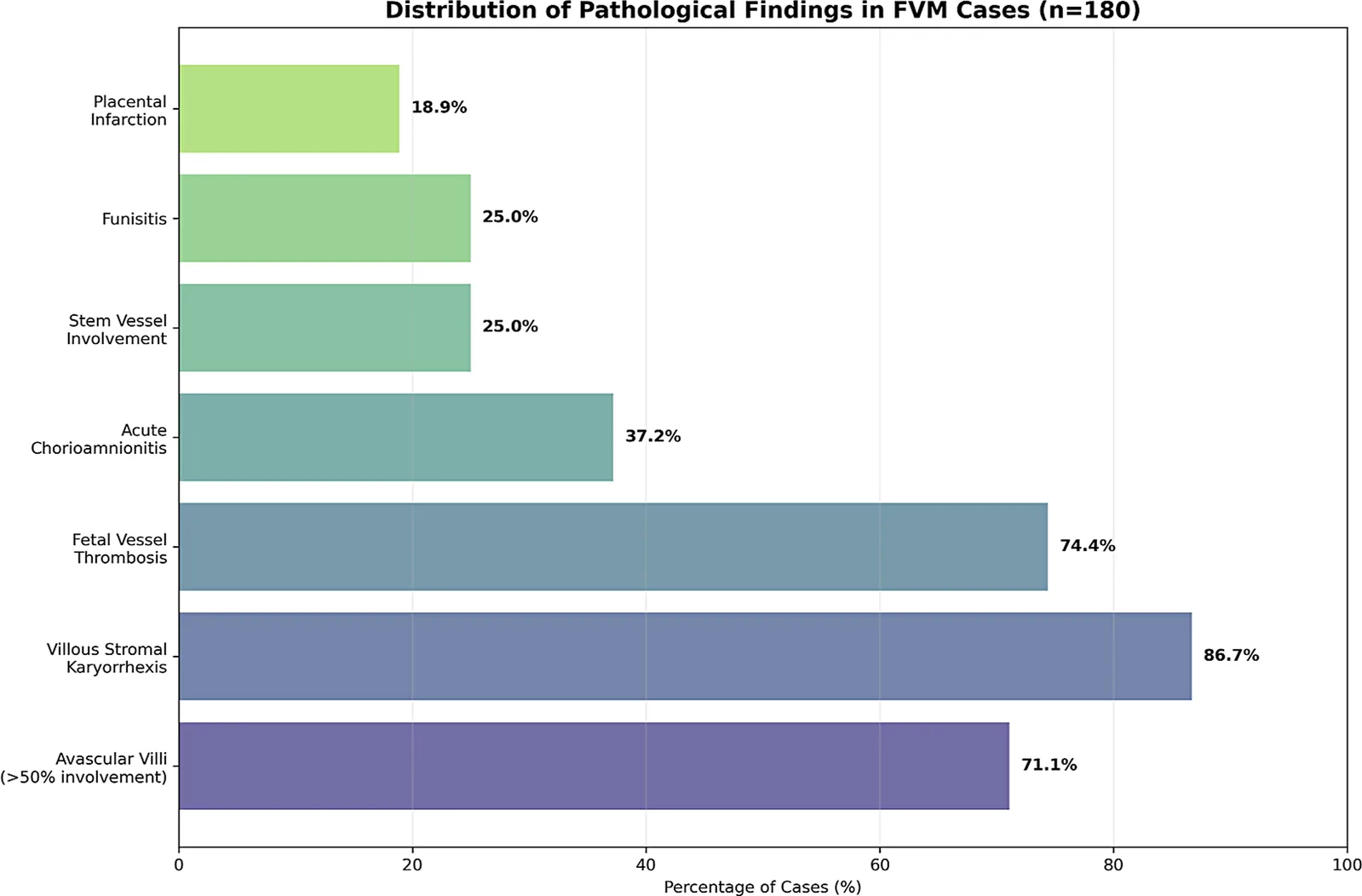
Distribution of pathological findings in FVM cases.

MVM, indicating maternal vascular malperfusion, was identified in 23 cases (12.8%). The coexistence of FVM and MVM, representing compromised maternal and fetal circulation, was associated with particularly severe clinical outcomes. Additional pathological findings included placental infarction in 34 cases (18.9%), retroplacental hematoma in 12 cases (6.7%), and villous edema in 78 cases (43.3%). Meconium-associated changes were present in 23 cases (12.8%), reflecting fetal distress patterns.

### Trimester-Specific Analysis

Trimester-specific analysis revealed distinct patterns of FVM presentation and associated pathology. First-trimester cases (*n* = 8, 4.4%) demonstrated severe pathological features, with 87.5% showing high-grade FVM. These cases were predominantly associated with pregnancy loss and showed extensive villous involvement with minimal inflammatory changes. Second-trimester cases (*n* = 43, 23.9%) showed the highest rate of associated inflammatory pathology at 18.6% of total cases. This group demonstrated a bimodal distribution of FVM severity with both mild and severe forms represented. The association with inflammatory conditions suggests increased vulnerability to infectious or inflammatory insults during mid-pregnancy. Third-trimester cases (*n* = 129, 71.7%) represented the majority of diagnoses and showed diverse pathological patterns. Notably, MVM occurred predominantly in third-trimester cases (19 cases, 10.7% of total cohort), reflecting the cumulative nature of maternal vascular compromise. High-grade FVM was present in 79.8% of third-trimester cases.

### Maternal Age-Related Patterns

Analysis of FVM severity by maternal age revealed unexpected patterns that differ significantly from conventional age-related pregnancy risks (Fig. 5). The highest rate of high-grade FVM was observed in the 30–34 years age group (88.3%, 40/46 cases), followed by the 25–29 years group (82.4%, 42/51 cases). Interestingly, the youngest maternal age group (<25 years) showed a relatively lower rate of high-grade FVM (74.4%, 32/43 cases), while the oldest group (≥40 years) demonstrated intermediate rates (80.0%, 8/10 cases). This inverted U-shaped distribution challenges conventional understanding of age-related pregnancy risks and suggests complex interactions between maternal age, genetic factors, and environmental influences specific to this population. The 35–39 years age group showed 76.7% high-grade FVM (23/30 cases), which was lower than the peak observed in younger age groups. This pattern may reflect selection bias, genetic factors, or differences in pregnancy management strategies across age groups.

**Fig. 5.**
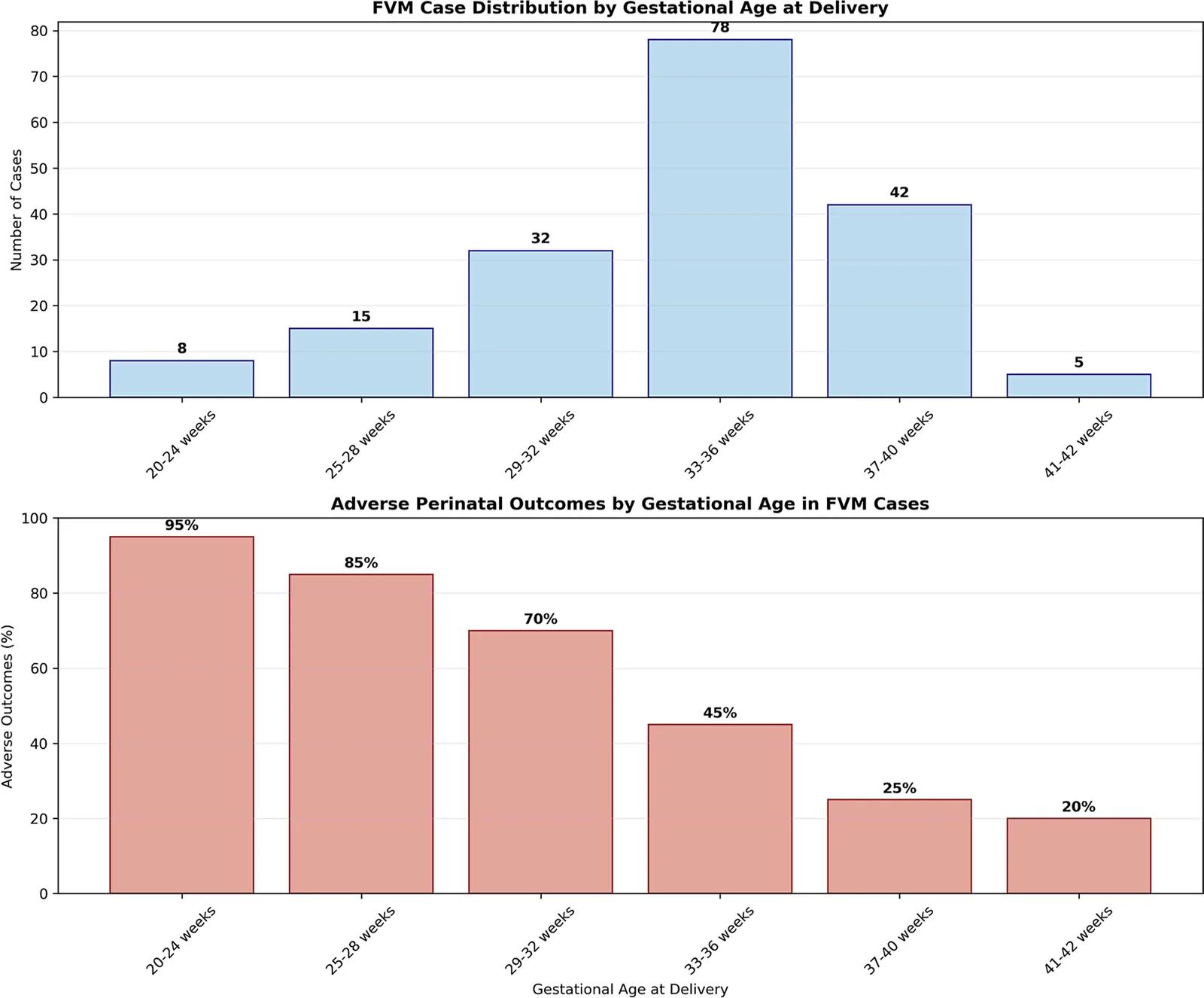
FVM case distribution by gestational age at delivery.

### Multiple Gestation Analysis

Twin pregnancies demonstrated significantly higher pathological burden compared to singleton pregnancies. High-grade FVM was present in 88.9% of twin pregnancies (16/18) compared to 79.5% of singleton pregnancies (128/162), representing a statistically significant difference (*p* = 0.041). In all of the examined placentas from dichorionic twin pregnancies, monochorionic twin pregnancies showed particularly severe pathological features, with 90.9% (10/11) demonstrating high-grade FVM compared to 85.7% (6/7) of dichorionic twins. The shared placental circulation in monochorionic twins appears to amplify the impact of vascular compromise, leading to more severe, no pathology was present in the second placenta, and no perinatal problems were seen in the second fetus. Associated pathological findings were also more frequent in twin pregnancies, with inflammatory pathology present in 66.7% of cases compared to 47.5% in singletons. MVM was observed in 38.9% of twin pregnancies compared to 9.2% in singletons.

### Clinical Outcomes and Correlations

Clinical outcome analysis revealed significant associations between FVM severity and perinatal morbidity. High-grade FVM cases demonstrated lower mean birth weights (2,456 ± 678 g vs. 2,789 ± 543 g in low-grade cases, *p* < 0.001) and higher rates of fetal growth restriction (34.7% vs. 16.7%, *p* = 0.032). Apgar scores at 5 min were significantly lower in high-grade FVM cases (mean 7.8 ± 1.4 vs. 8.6 ± 0.9 in low-grade cases, *p* < 0.001). Neonatal intensive care unit admission rates were higher in high-grade cases (41.7% vs. 22.2%, *p* = 0.028), reflecting increased neonatal morbidity. Stillbirth rates were elevated in cases with high pathology severity scores, with 13.3% of high-risk score cases resulting in stillbirth compared to 3.4% in low-risk cases (*p* = 0.045).

### Association between FVM Grade and Fetal Gestational Outcome

Comparison of gestational history of the fetus with FVM grade demonstrated a statistically significant association between these variables (Fig. 6). Overall, high-grade FVM was present in 144 of 180 cases, while low-grade FVM was present in 36 cases. The highest proportions of high-grade FVM were observed in fetuses with abnormal gestational history abortion (ABN) and intrauterine fetal death (IUFD), where high-grade FVM accounted for 26/29 cases (89.7%) and 89/101 cases (88.1%), respectively. In contrast, high-grade FVM was less frequent among cases with intrauterine growth restriction (IUGR) and normal fetal history, occurring in 16/28 cases (57.1%) and 13/22 cases (59.1%), respectively. The overall chi-square test showed a significant relationship between fetal gestational history and FVM grade, *p* = 0.0001. Pairwise Fisher’s exact testing with Bonferroni correction showed significant differences between ABN and IUGR *p* = 0.0423, IUFD and IUGR *p* = 0.0035, and IUFD and normal fetal history, *p* = 0.0172. No significant difference was found between ABN and IUFD, indicating that these two groups had similarly high proportions of high-grade FVM. Similarly, IUGR and normal fetal history did not differ significantly. For fetal gestational history, there were 4 groups, so this corresponds to 3 model parameters if used as a categorical predictor. Using high-grade FVM as the event, there were 144 events, resulting in EPV = 144/3 = 48.Using low-grade FVM as the event, there were 36 events, resulting in EPV = 36/3 = 12. So, the minority-class EPV is 12, which is generally acceptable for a simple logistic regression model with this single categorical predictor.

**Fig. 6.**
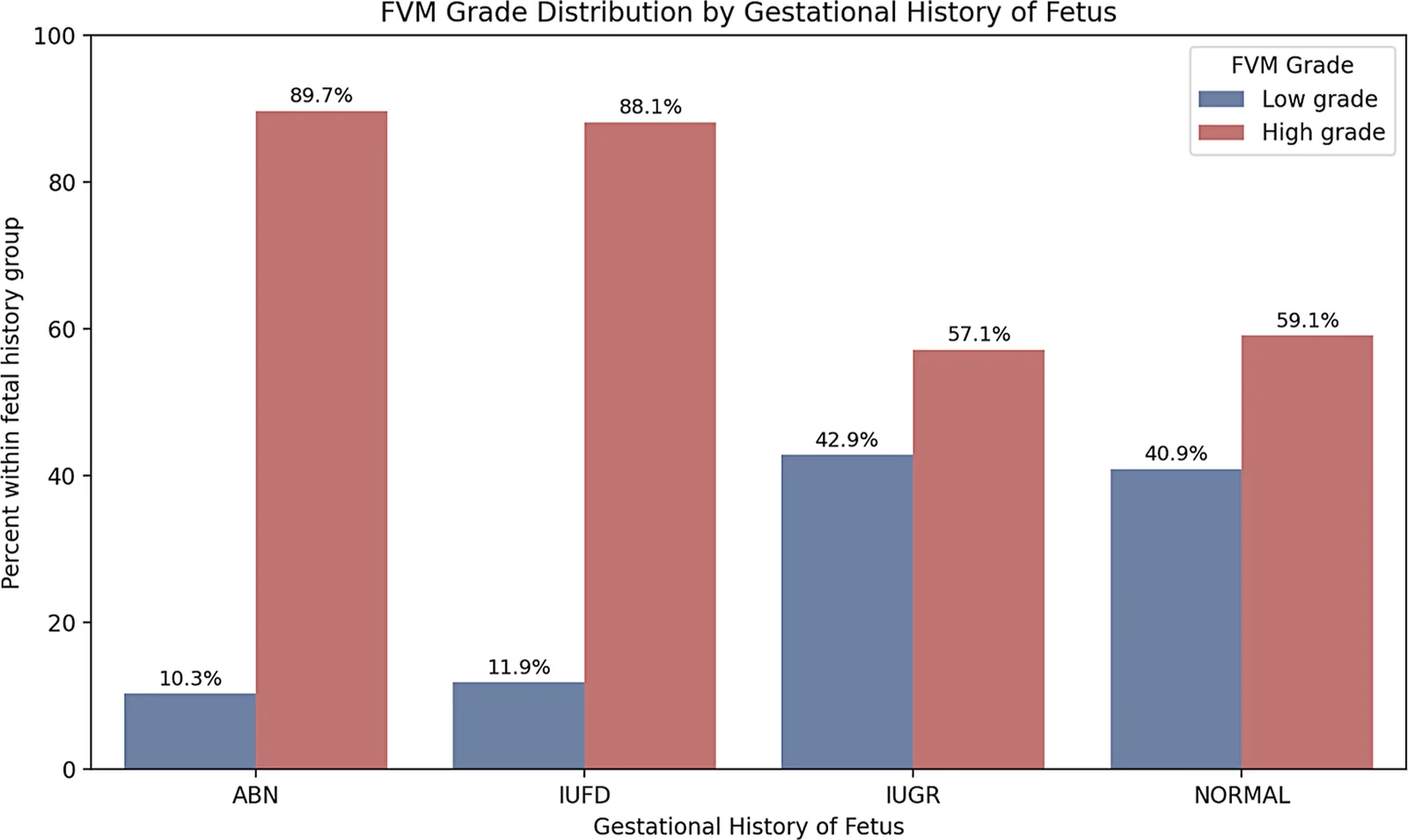
FVM severity and fetal outcome.

## Discussion

### Principal Findings and Population-Specific Patterns

This comprehensive 10-year analysis represents the first large-scale study of FVM in a Middle Eastern population, revealing several distinctive patterns that differ significantly from previously reported Western cohorts. First-trimester cases, while representing a small proportion of the cohort (4.4%), demonstrated severe pathological features, with 87.5% showing high-grade FVM. This finding suggests that early-onset FVM represents a particularly severe form of the condition, often associated with pregnancy loss and requiring specialized management approaches.

### Maternal Age-Related Risk Patterns

The unexpected inverted U-shaped distribution of FVM severity by maternal age. The predominance of high-grade FVM (80.0%) in our population substantially exceeds rates reported in European and North American studies, where high-grade FVM typically accounts for 50–60% of cases [14, 15]. This finding suggests either increased susceptibility to severe vascular compromise in Middle Eastern populations or differences in diagnostic thresholds and clinical practices. The overall FVM prevalence of 0.20% in our cohort aligns with international reports, indicating consistent recognition of this pathological entity across different healthcare systems and populations [16]. However, the severity distribution and associated pathological patterns demonstrate unique characteristics that warrant careful consideration in clinical practice and research applications.

### Trimester-Specific Pathological Patterns

The identification of distinct trimester-specific FVM patterns provides important insights into the temporal evolution of placental vascular pathology. The exclusive occurrence of MVM in third-trimester cases (12.8% of the total cohort) reflects the cumulative nature of maternal vascular compromise and suggests that combined fetal and maternal vascular pathology represents a particularly severe form of placental dysfunction. The high rate of inflammatory pathology in second-trimester cases (18.6%) may reflect increased vulnerability to infectious or inflammatory insults during mid-pregnancy, when placental development is most active and maternal immune adaptations are evolving. This pattern has important implications for pregnancy monitoring and intervention strategies, suggesting that enhanced surveillance for inflammatory conditions may be warranted during the second trimester in high-risk populations.

Analysis of FVM severity by maternal age revealed unexpected patterns that differ significantly from conventional understanding of age-related pregnancy risks. The peak risk in the 30–34 years age group (88.3% high-grade FVM) differs markedly from typical obstetric complications that show linear increases with advancing maternal age. This unique pattern may reflect complex interactions between genetic factors, environmental exposures, and reproductive health behaviors specific to Middle Eastern populations. The high rate of consanguineous marriages in Kuwait (estimated at 30–40% of marriages) may contribute to increased genetic susceptibility to vascular pathology in certain age groups [17, 18]. Alternatively, this pattern may reflect differences in pregnancy planning, prenatal care utilization, or lifestyle factors across age groups. Women in the 30–34 years age group may represent a population with specific risk factor combinations that predispose to severe FVM, including career-related stress, delayed childbearing, or specific environmental exposures. The relatively lower risk in the youngest age group (<25 years) may reflect protective factors associated with younger maternal age, including better vascular health, lower rates of chronic medical conditions, and different genetic expression patterns. However, this finding requires validation in larger cohorts and mechanistic studies to understand the underlying biological basis.

### Multiple Gestation and Vascular Pathology

The significantly higher pathological burden in twin pregnancies (88.9% vs. 79.5% singletons; high-grade FVM, *p* = 0.041) confirms the increased vulnerability of multiple gestations to placental vascular pathology. The shared placental circulation in monochorionic twins appears to amplify the impact of vascular compromise, leading to more severe pathological changes and clinical consequences. This finding has important implications for the management of twin pregnancies, suggesting that enhanced placental surveillance and earlier delivery may be warranted in cases with suspected FVM. The development of twin-specific risk assessment tools and management protocols should incorporate these findings to optimize perinatal outcomes. The higher rate of associated inflammatory pathology in twin pregnancies (66.7% vs. 47.5%) suggests increased susceptibility to infectious or inflammatory complications, possibly related to increased placental mass, altered immune responses, or mechanical factors associated with multiple gestation.

### Pathophysiological Considerations

The high prevalence of villous stromal karyorrhexis (86.7%) in our cohort indicates widespread acute fetal hypoxia, suggesting that FVM in this population frequently represents an acute or subacute process rather than chronic adaptation. This finding has implications for the timing of clinical interventions and the potential for reversibility of pathological changes (Fig. 7). The frequent coexistence of FVM with MVM (12.8% of cases) represents a particularly severe form of placental pathology involving both fetal and maternal vascular compartments. These cases manifested the worst clinical outcomes, supporting the concept that combined vascular pathology represents a distinct high-risk category requiring specialized management. The presence of complex thrombosis patterns in 37.2% of cases (Fig. 8, 9) suggests underlying hypercoagulable states or genetic predispositions that may be more prevalent in Middle Eastern populations. Investigation of thrombophilia markers and genetic polymorphisms affecting coagulation pathways may provide insights into population-specific risk factors.

**Fig. 7.**
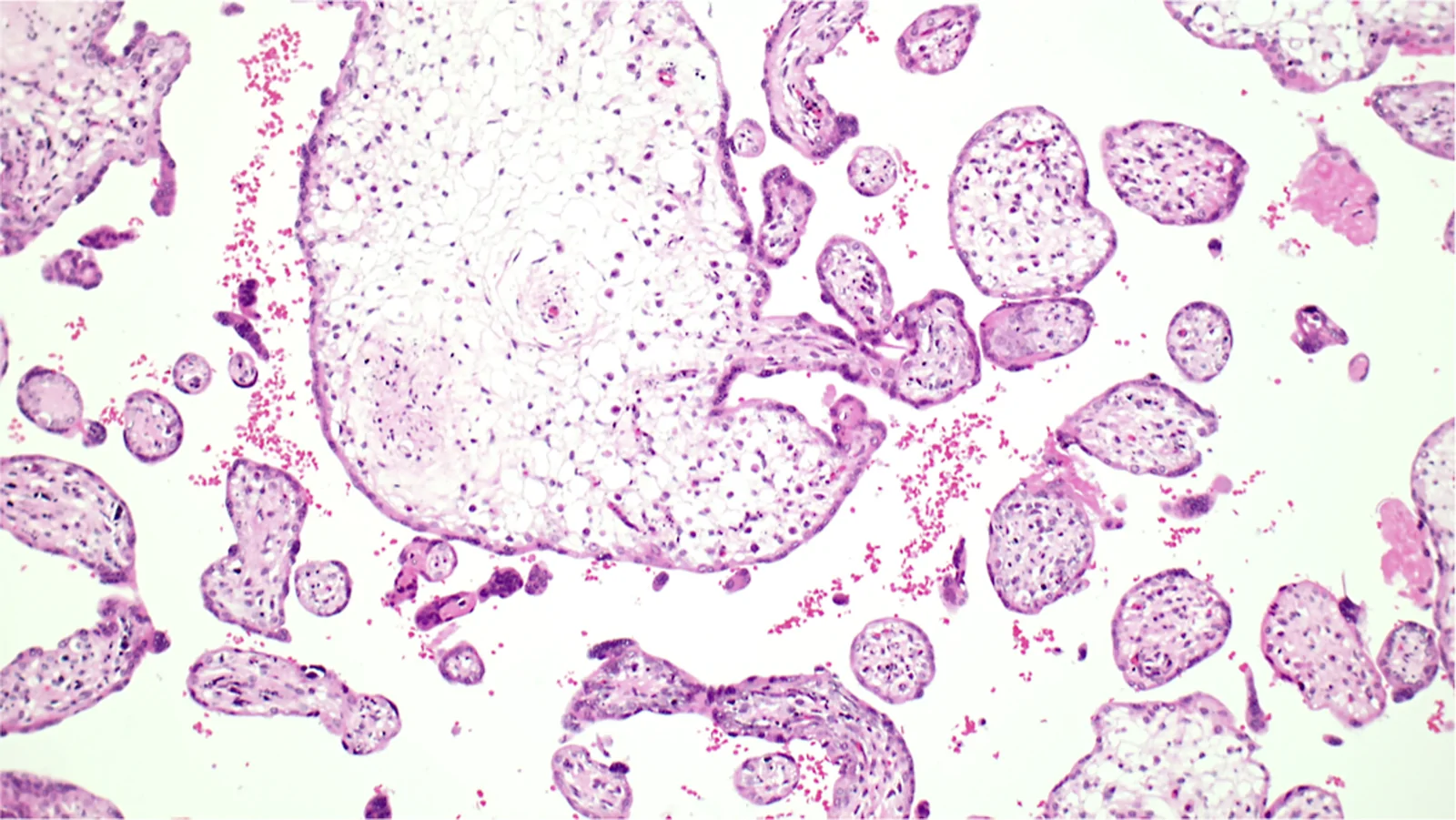
Placental villous karyorrhexis in a 28-week-old fetus.

**Fig. 8.**
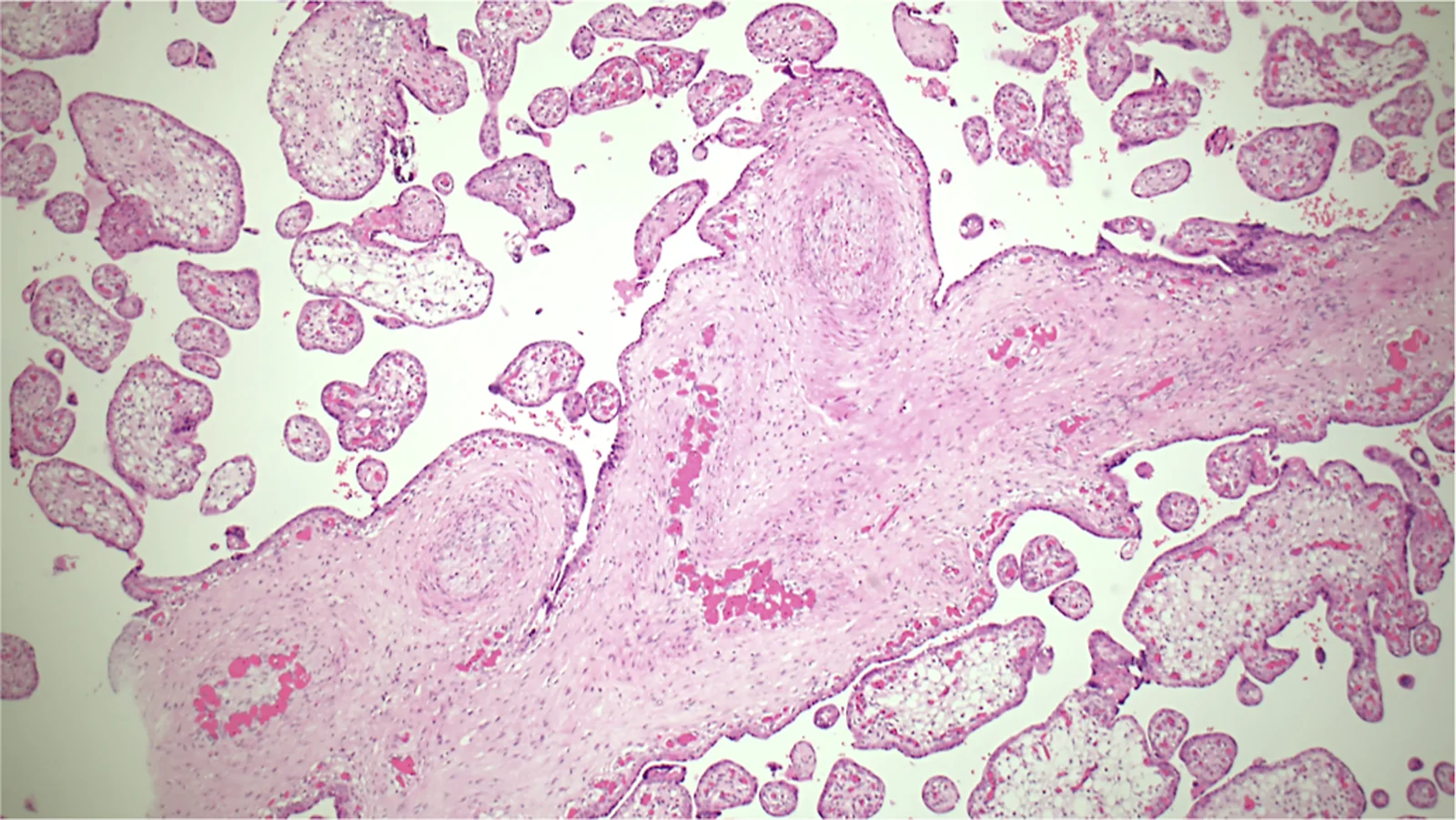
Various stages of villous vascular thrombosis in the stem villi of a 30-week-old placenta.

**Fig. 9.**
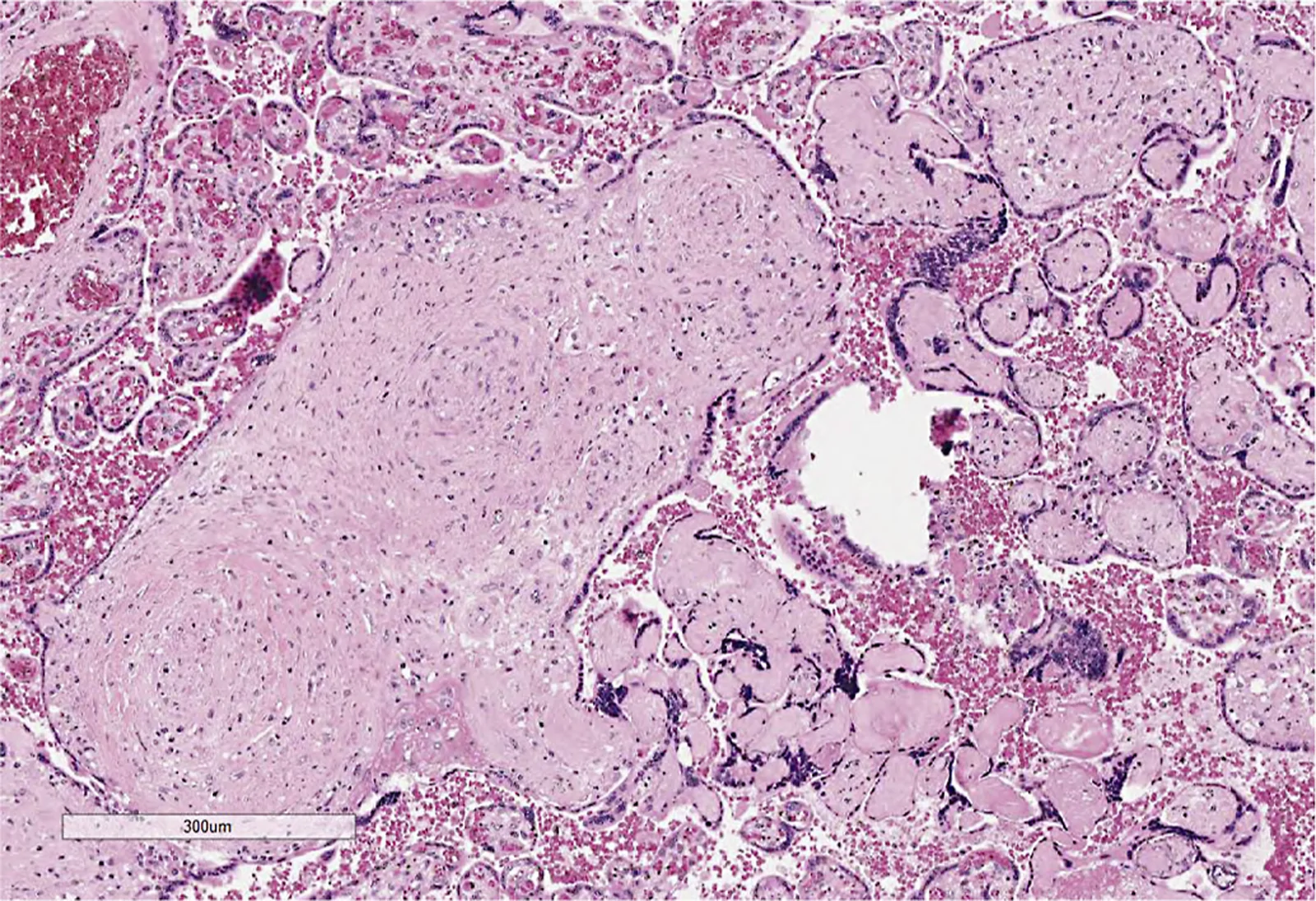
Complete thrombosis of stem villi vessels and downstream fibrosis of all terminal villi supplied by it.

### FVM Grade and Fetal Gestational Outcome

These findings suggest that higher-grade FVM is more strongly associated with adverse fetal outcomes, particularly IUFD and abnormal fetal gestational history. The predominance of high-grade FVM in the IUFD and ABN groups supports the interpretation that more severe fetal vascular compromise may be linked to clinically significant fetal injury or demise. In contrast, the lower proportion of high-grade FVM in IUGR and normal fetal history cases may indicate that growth restriction and otherwise normal fetal outcomes can occur across a broader spectrum of FVM severity, including low-grade lesions. The lack of significant difference between ABN and IUFD suggests that both groups may represent a similar high-risk pathological pattern, whereas the similarity between IUGR and normal cases may reflect either milder vascular involvement or additional non-FVM contributors to fetal growth restriction. Overall, these results support the potential clinical relevance of FVM grading, particularly in distinguishing cases associated with severe adverse fetal outcomes; however, interpretation should consider the retrospective nature of the dataset and the relatively smaller sample sizes in some subgroups, especially ABN, IUGR, and normal fetal history.

### Limitations and Future Directions

Several limitations should be acknowledged in interpreting these findings. The retrospective design limits the ability to establish causal relationships and may introduce selection bias toward more severe cases requiring placental examination. The single-center design, while providing consistency in diagnostic approaches, may limit generalizability to other Middle Eastern populations or healthcare settings. The lack of long-term neurodevelopmental outcome data represents a significant limitation, as emerging evidence suggests important associations between placental vascular pathology and childhood developmental outcomes. Future prospective studies should incorporate long-term follow-up to assess the full clinical impact of FVM in this population. The absence of detailed genetic analysis limits our understanding of population-specific susceptibility factors. Future studies should incorporate genetic screening for thrombophilia markers, inflammatory gene polymorphisms, and other relevant genetic variants that may contribute to FVM risk in Middle Eastern populations.

### Clinical Recommendations

Based on these findings, we propose several clinical recommendations for the management of FVM in Middle Eastern populations. Enhanced surveillance protocols should be implemented for high-risk groups, including twin pregnancies, cases with maternal age of 30–34 years, and pregnancies with inflammatory complications during the second trimester. The research priorities should include prospective studies with long-term follow-up, genetic analysis of susceptibility factors, and investigation of preventive interventions for high-risk populations. International collaboration with other Middle Eastern centers would enhance the generalizability of findings and support the development of region-specific management guidelines.

## Conclusions

This study provides the first comprehensive analysis of FVM patterns in a Middle Eastern population, revealing distinct characteristics that differ from Western populations. The predominance of high-grade FVM, unique age-related risk patterns, and trimester-specific pathological profiles support the need for population-specific approaches to FVM assessment and management. The clinical implications of these findings extend beyond immediate perinatal care to include long-term health outcomes and public health planning. Continued research in this area will enhance our understanding of FVM pathophysiology and support the development of targeted interventions to improve maternal and fetal outcomes in Middle Eastern populations.

## Acknowledgments

The authors thank the staff of the Department of Pathology and the Department of Obstetrics and Gynecology at the Maternity Hospital, Kuwait, for their support in data collection and case identification. We acknowledge the contribution of the medical records department for facilitating access to clinical data. Special thanks to the laboratory technicians who performed the histological preparations and the nursing staff who assisted with specimen collection and processing. Approval to perform the project was given by the Ministry of Health of the State of Kuwait.

## Statement of Ethics

This study was performed in accordance with the Declaration of Helsinki and was approved by Kuwait University’s College of Medicine Ethics Committee and the Ministry of Health Ethics Committee (No. VDR/EC/3725 and 3150, respectively).

## Conflict of Interest Statement

The authors declare no conflicts of interest.

## Funding Sources

This research received no specific grant from any funding agency in the public, commercial, or not-for-profit sectors.

## Author Contributions

The sole author, Salah Al-Waheeb, was involved in collecting the data, reviewing the pathology slides, data analysis and writing the manuscript.

## Data Availability

The data used in this manuscript are embedded with the manuscript.
